# A Prospective Single-Center Brazilian Study Investigating the Efficacy and Safety of Prophylactic Phenylephrine Infusion for the Management of Hypotension During Cesarean Section Under Spinal Anesthesia

**DOI:** 10.7759/cureus.42156

**Published:** 2023-07-19

**Authors:** Marcio L Benevides, Breno Wilson B Andrade, Henry Manfried D Zambardino, Marco Antonio M Benevides

**Affiliations:** 1 Department of Anesthesiology, Hospital Geral e Maternidade de Cuiabá, Cuiaba, BRA; 2 Department of Anesthesiology, Hospital Geral e Maternidade de Cuiabá, Cuiabá, BRA; 3 Medicine, Universidade de Cuiabá, Cuiabá, BRA

**Keywords:** phenylephrine, maternal outcomes, fetal outcomes, hypotension, spinal anesthesia, cesarean section

## Abstract

Background

Maternal hypotension occurs in up to 80% of parturients during cesarean section (CS) under spinal anesthesia. Phenylephrine, a direct-acting α-1 agonist, has been widely recommended for the prevention of hypotension. We evaluated the efficacy and safety of phenylephrine infusion to prevent hypotension in obese and non-obese patients during cesarean section.

Methods

One hundred forty-one patients were included in this single-arm study. Patients received prophylactic phenylephrine infusion at a rate of 50 μg/min^-1^ immediately after spinal local anesthetic injection until delivery. Hypotension was defined as a systolic blood pressure <100 mmHg or <20% of baseline. The primary outcome was the incidence of hypotension.

Results

The incidence of hypotension was 17%. The median and interquartile range (IQR) of the number of hypotensive episodes was 0 (0-0). It was observed that 79.1% of the patients had hypotension in the first six minutes. Reactive hypertension and bradycardia occurred in 20.5 and 12.7% of the patients, respectively. In addition, there was a higher incidence of bradycardia in pregnant women with a body index mass of < 30 kg/m^-2^. Patients with baseline systolic blood pressure <120 mmHg had a threefold increased risk of hypotension. The incidence of nausea and vomiting was 13.4 and 2.8%, respectively. The incidence of an Apgar score <7 at the first minute was 2.8%, and no neonates presented an Apgar score <7 at the fifth minute. A pH of <7.2 occurred in 6.3% of the neonates. All neonates had no sequelae and were discharged together with their mothers.

Conclusion

The prophylactic infusion of phenylephrine 50 μg/min^-1^ is safe and demonstrates efficacy in reducing maternal hypotension providing adequate maternal hemodynamic stability during CS under spinal anesthesia.

## Introduction

Spinal anesthesia is the most commonly used anesthetic technique for cesarean section (CS) because of its simplicity, cost-effectiveness, and rapid onset of action, and it offers reliable surgical anesthesia with a low failure rate avoiding general anesthesia [1]. However, spinal anesthesia‐induced hypotension remains an important problem. The absence of labor, a positive supine stress test, hypovolemia, high preoperative anxiety, a high baseline heart rate, increased heart rate variability, a recent occurrence of supine intolerance or supine hypotensive syndrome, and a peak sensory block height >T4 dermatome are some of the risk factors for hypotension [2,3]. However, hypotension occurs in up to 80% of parturients, even if the prediction of hypotension could alter management and facilitate early intervention [3]. Hypotension may cause dizziness, nausea, and vomiting with a risk of bronchoaspiration of gastric contents, cardiovascular collapse, loss of the mother's consciousness, and reduced uteroplacental flow with consequent fetal acidosis [4]. Therefore, the prevention of this complication has been of great importance for anesthesiologists and obstetricians. Preventive measures include pre- or coadministration of intravenous fluids, use of ondansetron before spinal anesthesia, use of lower doses of local anesthetic in spinal anesthesia, lateral displacement of the gravid uterus, lower limb compression, and use of vasopressors [5].

Aorto-caval compression by the uterus and venodilation leading to decreased venous return and cardiac output was previously considered the main mechanism of hypotension after spinal anesthesia during CS. However, a reduction in systemic vascular resistance (vasodilation of small arterioles) has been shown to be the most important mechanism [4]. Therefore, the use of vasopressors, especially when used prophylactically, is useful and should be used routinely unless contraindicated [4-6]. 

The most commonly used vasopressors are ephedrine, metaraminol, and phenylephrine [7]. Phenylephrine, a direct-acting α-1 agonist, has been widely recommended for the prevention and treatment of maternal hypotension during CS under spinal anesthesia, and its use is associated with a lower rate of fetal acidosis compared to ephedrine [7]. Additionally, the use of phenylephrine for the prevention of hypotension caused by the high neuraxial block is an important approach to lower insurance claims that can lead to serious medico-legal implications [8].

The objective of this study was to evaluate the efficacy and safety of phenylephrine infusion to prevent hypotension in obese and non-obese patients during CS.

## Materials and methods

Subjects and ethics

Institutional Ethics Committee for Human Research approved this study on December 15, 2020 (protocol number: 08047918.0.0000.5165), and it was registered in the Brazilian Clinical Trials database (RBR-2tnnhvg). We obtained written, informed consent from all patients.

Inclusion criteria and exclusion criteria

Pregnant women 18 years and older of the American Society of Anesthesiologists graded II and III undergoing elective CS under spinal anesthesia were eligible to participate in this prospective single-arm study. Exclusion criteria included gestational age of <37 weeks, pregnancy with multiple fetuses, labor, preeclamptic, chronic hypertension with arterial blood pressure >140/90 mmHg, New York Heart Association functional class >II, cerebrovascular disease, intrauterine growth retardation, fetal malformations, contraindications to spinal anesthesia, failure of spinal anesthesia, and conversion to general anesthesia. 

Anesthesia, phenylephrine infusion, and operative technique

In the operating room, monitoring was conducted through electrocardioscopy, noninvasive blood pressure suitable for each patient's arm circumference, and pulse oximetry throughout the anesthetic surgical procedure. Three readings of systolic blood pressure (SBP) and heart rate (HR) were recorded every two minutes in a sitting position, and the average of these readings was taken as the baseline value.

An 18 G intravenous (IV) cannula was inserted, 500 mL of lactated Ringer's solution and 2 g of cefazolin were administered before the subarachnoid puncture, followed by 10 mL/kg/h^-1 ^of lactated Ringer's solution until the end of the operation. Spinal anesthesia was performed using a 25-G x 9-mm or 25-G x 12-mm Quincke needle at the L2-3 or L3-4 vertebral interspace with 0.5% hyperbaric bupivacaine 12.5 mg combined with morphine 0.1 mg with the patient in the sitting position [9,10]. All the patients received a continuous infusion of phenylephrine 50 μg/min^-1^ (0.05 mg/mL^-1^) immediately after spinal anesthesia [9]. The phenylephrine infusion was connected to the IV line via a three-way stopcock and was administered at a rate of 60 mL/h until delivery [9]. The patient was placed in the horizontal supine position with left uterine displacement. The sensory block level was tested bilaterally by the loss of thermal sensation, and surgery was allowed to start if the level of sensory block was at least at the T6 dermatome [9]. All patients received an indwelling bladder catheterization. The operation was performed using the Pfannenstiel technique.

Blood pressure and HR were recorded at two-minute intervals until delivery and at the fifth minute after delivery. Hypotension was defined as a decrease in SBP of 20% or less than 100 mmHg of baseline value [11] and was treated with a bolus of phenylephrine 0.1 mg IV. The primary outcome was the incidence of hypotension. Hypotension was treated with a bolus of phenylephrine 0.1 mg IV. Reactive hypertension was defined as an increase in SBP >120% from baseline. Once hypertension occurred, the infusion of phenylephrine was discontinued, and it was restarted only when the SBP decreased to <120% of baseline [9]. If bradycardia (defined as HR <50 beats/min^-1^) occurred [9], atropine 0.5 IV was administered, and ondansetron 4 mg IV was administered if nausea or vomiting occurred not related to arterial hypotension [8]. Immediately after delivery, 3 IU of oxytocin was administered as a bolus over three seconds, followed by 12 IU as a slow infusion [9].

Outcome variables

Data were collected regarding the variables: age, weight, height, body mass index (BMI), parity, gestational age, comorbidities, previous CS, fasting time, baseline SBP, baseline HR, sensory block level reached 15 minutes after subarachnoid injection, the interval of time from the spinal drug injection to the skin incision (spinal-to-incision interval), the interval of time from skin incision to delivery (incision-to-delivery interval), neonatal weight, and fluids infused during surgery.

The primary outcome of the study was the incidence of hypotension. Secondary outcomes were the number of hypotensive episodes (the number of times hypotension occurred until delivery), rescue phenylephrine bolus (μg) used for hypotension treatment, the time to first episode of hypotension, the incidence of reactive hypertension, the incidence of bradycardia, the incidence of nausea and vomiting, Apgar scores of <7 at the first and fifth minutes, pH, pO_2_, pCO_2_, and base excess of the umbilical artery.

Sample size calculation and data analysis

The sample size calculation was based on the incidence of hypotension of 60% found in the pilot study. A margin of error of 10% and a confidence level of 95% were considered. Thus, it was necessary to include at least 93 parturients in this study. Assuming there was approximately a 20% loss, 115 participants were expected to be enrolled.

Descriptive statistics are presented as numbers (percentages), medians and interquartile ranges (IQRs), and means ± standard deviations (SDs). Differences in incidence and 95% confidence interval (95% CI), odds ratio (95%CI), median, and mean of outcomes between groups with BMI < 30 and ≥ 30 kg/m^-2^ were analyzed. The chi-square test was used to analyze categorical data. The Student's t-test was used to analyze continuous, normally distributed data. The Mann-Whitney test was used to analyze discrete or continuous data without a normal distribution. Multiple logistic regression was applied for the analysis of potential predictive factors for maternal hypotension. The exposition variables included in the model presented a p-value <0.2 in the univariate analysis. For all analyses, a p-value <0.05 was regarded as statistically significant.

## Results

**Figure 1:**
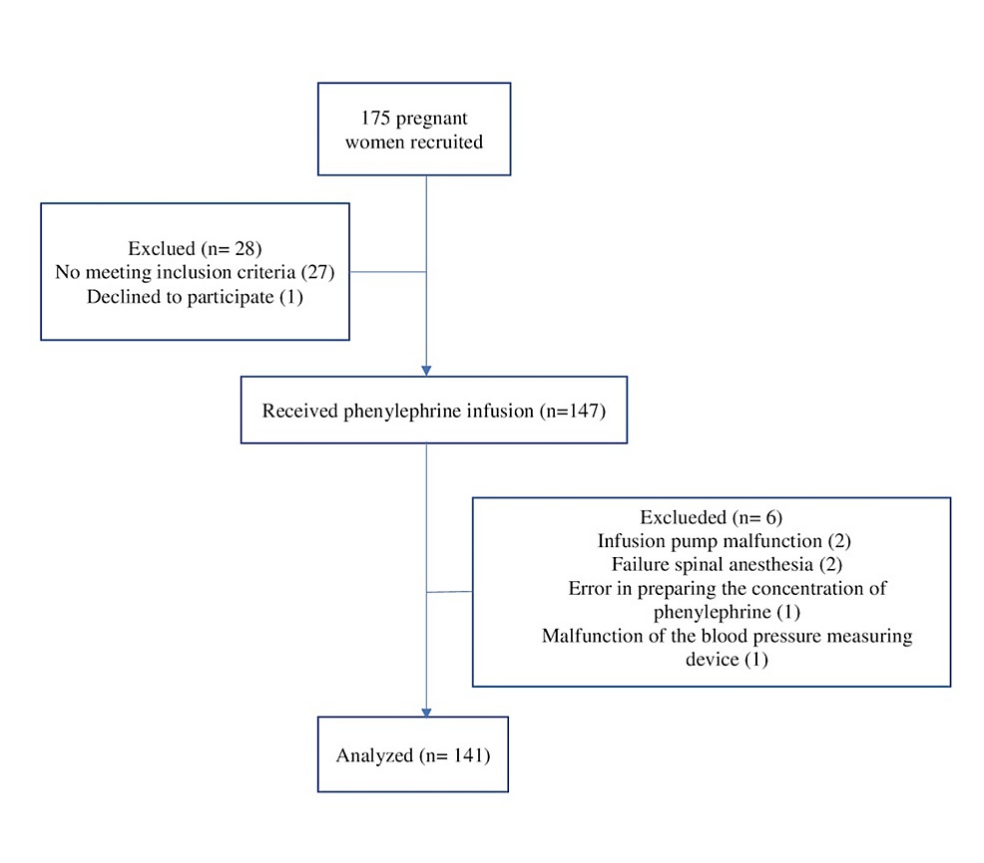
The patient flow diagram of the study

One hundred and seventy-five patients were recruited for the study. Twenty and eight patients did not meet the inclusion criteria or declined to participate. One hundred and forty-seven patients received the intervention. After exclusions, data analysis was performed on 141 patients. The patient flow diagram of the study is shown in Figure 1.

The mean ± SD of maternal age in years was 29.9 ± 6.5. Ninety-six patients (68.1%) had a BMI ≥30 kg/m^-2^. Ninety-seven patients (68.8%) had previously undergone one or more cesarean sections. Fasting times for liquids and solids were 11.4 ± 4.5 and 13 ± 4.6 hours, respectively. The level of sensory block at T4 to T6 after 15 minutes occurred in 97.8% of parturients. The spinal-to-incision interval was 12.1 ± 3.9 minutes, and 19 (13.5%) of the patients had a 90th percentile of the interval between incision-to-delivery ≥ 16 minutes. The patient characteristics and intraoperative variables are shown in Table 1.

**Table 1:** Patient characteristics and intraoperative variables BMI - body mass index, SBP - systolic blood pressure, HR - heart rate Data are expressed as the mean ± standard deviation and n (%); * 90th percentile of the incision-to-delivery interval

Variables	(n= 141)
Age (years)	29.9 ± 6.5
Weight (kg)	85.8 ± 15.3
Height (cm)	161.4 ± 6.8
BMI at delivery (kg/m^-2^)	33 ± 5.8
BMI <30	45 (31.9)
BMI ≥30	96 (68.1)
Gestational age (weeks)	38.9 ± 1.5
Parity	
0	24 (17)
1 to 2	89 (63.1)
≥ 3	28 (19.9)
Previous cesarean section	
0	44 (31.2)
1 to 2	89 (63.1)
≥ 3	28 (19.9)
Comorbidity	
Gestational diabetes mellitus	15 (10.6)
Chronic hypertension	8 (5.6)
Others	11 (7.8)
Baseline SBP (mmHg)	130.9 ± 14.3
Baseline HR (beats/min^-1^)	89 ± 16
Fasting for clear liquids (hours)	11.4 ± 4.5
Fasting for solids (hours)	13 ± 4.5
Sensory block level (dermatome)	
< T4	13 (2.2)
T4–T6	138 (97.8)
Spinal-to-incision interval (min)	12.1 ± 3.9
Incision-to-delivery interval (min)	10.1 ± 4.4
Incision-to-delivery interval ≥ 16 min*	19 (13.5)
Neonatal weight (g)	3463 ± 467
Fluids infused during surgery (mL)	1569 ± 253

The incidence of maternal hypotension was 17% (95% CI: 11.7-24.8). The median and IQR of the number of hypotensive episodes were 0 (0-0). In 20.5% of the patients, reactive hypertension occurred, requiring interruption of the phenylephrine infusion, but there was more than one interruption in only 4.9%. A healthy 18-year-old patient had a headache with an SBP of 180 mmHg in the fourth minute (the previous measurement in the second minute was an SBP of 132 mmHg) but promptly recovered after discontinuation of the phenylephrine infusion. Eighteen pregnant women (12.7%) had bradycardia. The incidence of nausea and vomiting was 13.4 and 2.8%, respectively. Four neonates (2.8%) had Apgar scores of <7 at the first minute; however, no neonate presented Apgar scores of <7 at the fifth minute. Nine (6.3%) neonates had a pH of <7.2. All neonates had no sequelae and were discharged together with their mothers. The obstetric and neonatal outcomes are shown in Table 2.

**Table 2:** Obstetric and neonatal outcomes Data expressed: mean ± standard deviation; n, % (95% confidence interval); odds ratio (95% confidence interval); and median (interquartile range)

Variables	(n = 141)
Maternal hypotension	24, 17 (11.7 - 24.8)
Number of hypotensive episodes	0 (0-0)
Rescue phenylephrine bolus (µg)	31.2 ± 98.6
Reactive hypertension	29, 20.5 (14.2- 28.1)
Interruption of phenylephrine infusion	
None	112 (79.4)
1	22 (15.6)
≥2	7 (4.9)
Bradycardia	18, 12.7 (7.7 - 19.4)
Nausea	19, 13.4 (8.3 – 20.2)
Vomiting	4, 2.8 (0.7 - 7.1)
pH	7.29 ± 0.06
pH <7.2	9, 6.3 (2.9 - 11.7)
pO_2_ (mmHg)	23.5 ± 11.8
pCO_2_ (mmHg)	48.8 ± 8.8
Base excess (mmol.l^−1^)	-3.2 ± 2.2
Apgar score <7 at first min	4, 2.8 (0.7 - 7.1)
Apgar score <7 at fifth min	0, 0 (0 - 0)

The time to the first episode of hypotension was 25.8 minutes. Twenty-four patients had hypotension within the first 18 minutes. Of these, 19 (79.1%) had hypotension within the first 6 minutes.

There was a higher incidence of bradycardia in pregnant women with a BMI < 30 kg/m^-2 ^than in those with a BMI ≥ 30 kg/m^-2^ (24.4 versus 7.2%, *p* = 0.004) and OR 4.11 (1.47-11.48). In all other comparisons, there was no significant difference. See Table 3. 

**Table 3:** Obstetric and neonatal outcomes according to body mass index (BMI) Data expressed: mean ± standard deviation and n, % (95% confidence interval); odds ratio (95% confidence interval); and median (interquartile range) NA - not applicable; *Chi-squared test

Variables	BMI < 30 Kg/m^-2^	BMI ≥ 30 Kg/m^-2^	OR (95% CI)	p-value
	(n = 45)	(n = 96)		
Maternal hypotension	5, 11.1 (3.3 - 21.8)	19, 19.7 (12.3 - 29.7)	0.50 (0.17 -1.45)	0.201
Number of hypotensive episodes	0 (0-0)	0 (0-0)	NA	0.352
Rescue phenylephrine bolus (µg)	13.3 ± 40.4	39.6 ± 115.5	NA	0.142
Reactive hypertension	13, 28.8 (16 - 44)	16, 16.6 (9.4 - 25.6)	2.03 (0.87 - 4.69)	0.097
Bradycardia	11, 24.4 (12.8 - 39.4)	7, 7.2 (2.9 - 14.4)	4.11 (1.47 - 11.48)	0.004*
Nausea	6, 13.3 (5.0 - 26.7)	13, 13.5 (7.4 - 22.0)	0.98 (0.34 - 2.77)	0.973
Vomiting	1, 2.2 (0.0 - 11.7)	3, 3.1 (0.6 - 8.8)	0.70 (0.07 - 6.96)	0.764
pH	7.30 ± 0.06	7.29 ± 0.06	NA	0.235
pH <7.2	2, 4.4 (0.5 - 15.1)	6, 6.2 (2.3 - 13.1)	0.69 (0.13 - 3.6)	0.667
pO_2_ (mmHg)	22.8 ± 8.8	24 ± 13.1	NA	0.733
pCO_2_ (mmHg)	47.9 ± 8.8	49.3 ± 8.8	NA	0.178
Base excess (mmol.L^−1^)	- 3.02 ± 2.1	- 3.3 ± 2.2	NA	0.973
Apgar score <7 at first minute	3, 6.6 (0.0 - 13.9)	1, 3.5 (0.0 - 8.4)	6.78 (0.68 - 67.15)	0.101
Apgar score <7 at fifth minute	0, 0 (0 - 0)	0, 0 (0 - 0)	0 (0 - 0)	-

All patient characteristics and intraoperative variables described in Table 1 were included in univariate analysis. In the multiple logistic regression, patients who presented a baseline SBP <120 mmHg had a threefold increased risk of hypotension (Table 4).

**Table 4:** Multivariate analysis of maternal hypotension predictors. OR - odds ratio, CI - confidence interval

Variables			Beta	Adjusted OR	CI (95%)	p-value
Baseline SBP <120 mmHg		1.125		3.080	1.075 - 8.828	0.036
Neonatal weight		0.001		1.001	1.000 - 1.002	0.061
Incision-to-delivery interval	0.086			1.090	0.967 - 1.228	0.157

## Discussion

In this study, we found that the incidence of hypotension was 17%. The median and interquartile range (IQR) of the number of hypotensive episodes was 0 (0-0). It was observed that 79.1% of the patients had hypotension in the first six minutes. Reactive hypertension and bradycardia occurred in 20.5 and 12.7% of the patients, respectively. There was a higher incidence of bradycardia in pregnant women with a body index mass of <30 kg/m^-2^. In addition, patients with baseline systolic blood pressure <120 mmHg had a threefold increased risk of hypotension.

The prevention of hypotension during cesarean section has fundamental importance, aiming at maintaining maternal and fetal well-being.

Meta-analyses [6] and guidelines [7] recommend that vasopressors should be used routinely and preferably prophylactically. International consensus, published in 2018, stated that "it seems preferable to start phenylephrine infusion at a rate of 25-50 μg/min^-1^, and titrate to response" [7]. We preferred to use phenylephrine at a fixed rate dose of 50 μg/min^-1 ^for practicality and because this dose has been shown to be effective and safe, as demonstrated in several other studies [12-15].

A dose of 12.5 mg of hyperbaric bupivacaine associated with 100 µg of morphine has been routinely used in spinal anesthesia for CS in our service. In the systematic review, the calculated effective dose of 95% of bupivacaine ranged from 8.8 mg to 15 mg, corroborating the choice of this dose of bupivacaine in the present study [10].

In the present study, the incidence of hypotension was 17%. We recognize that the prevention of hypotension in CS is multifactorial; however, the prophylactic infusion of phenylephrine seems to have a greater impact on the prevention of maternal hypotension, among other factors. Recently, in an observational study performed in our service using the same hyperbaric bupivacaine dosing but without the use of prophylactic phenylephrine, we found a high incidence of hypotension of 67.7% [16], showing that with the use of phenylephrine infusion occurred an approximately fourfold reduction in hypotension. Several studies have demonstrated similar results to the present study. Allen et al. reported a 15% incidence of hypotension [17]. Ortiz-Gómez et al. [12] reported an incidence of hypotension of 20.9%. George et al. [13] found a high incidence of maternal hypotension in obese pregnant women who received rescue bolus dosing of phenylephrine to treat hypotension (75%), in contrast to those patients who received a 50 mg/min^-1^ prophylactic phenylephrine (27%) [13]. Another study used preloading with a 5 mL/kg^-1 ^bolus of hydroxyethyl starch that was rapidly infused intravenously before the realization of the regional procedure and reported an incidence of 12.1% hypotension [15]. Interestingly, some studies reported very low or even no hypotension with the use of the same dose of phenylephrine used in the present study. In one of these, a lower dose of hyperbaric bupivacaine (10 mg) was used, and the incidence of hypotension was reported to be 2.5% [14]. In another study, 20 mL/kg^-1^ hydroxyethyl starch (limited to 1 liter) was infused until delivery, and no maternal hypotension was reported [18]. This absence of hypotensive events is perhaps the result of using a high dose of hydroxyethyl starch. However, there are concerns associated with colloid use in pregnant women [2], and there is on global trend of hydroxyethyl starch losing its approval for use due to mortality, acute kidney injury, and excess bleeding in surgical patients [19]. 

Reducing patients' exposure to hypotension over time is of fundamental importance. In this trial, the median and IQR of the number of hypotensive episodes was 0 (0-0). Two studies demonstrated a substantial reduction in the number of episodes of hypotension in patients who received a prophylactic infusion of phenylephrine when compared to those who received only a bolus of phenylephrine to treat hypotension: 0 (0-1) versus 3 (1-4) and 0 (0-0) versus 2 (1-3), both p<0.001 [13,17]. These results are consistent with our results.

It was observed that 79.1% of the patients had hypotension in the first six minutes. This may demonstrate the need to administer a bolus of phenylephrine immediately prior to initiation of a fixed rate of phenylephrine after spinal anesthesia to avoid the delay in achieving effective blood levels, as performed by Kuhn et al. [20].

Reactive hypertension appears to be dose-dependent, as demonstrated in the work of Xiao et al. [21]. In the present study, 20.5% (29/141) of the patients had at least one episode of reactive hypertension. Discrepant results have been reported with the use of phenylephrine infusion at a rate of 50 mg/min^-1^, with an incidence of reactive hypertension ranging from 7.3 to 44% [13-15]. However, this undesirable effect may be rectified by a reduction or stop in the infusion rate.

The prophylactic administration of phenylephrine infusion constitutes a first-line drug to manage hypotension after spinal anesthesia, but its use can lead to dose-dependent reflex bradycardia and decreased cardiac output. In this scenario, the interruption of the phenylephrine infusion may be sufficient to solve these problems. However, in a few situations, bradycardia may occur with hypotension requiring the administration of a vasopressor acting on α-1 and beta-1 agonist receptors, with or without anticholinergics [4]. Such agents could be norepinephrine which has been shown recently to be associated with a much lower incidence of maternal bradycardia [22]. A recent review (2020) reported that phenylephrine caused maternal bradycardia when compared with placebo, odds ratio and 95% CI: 0.23 (0.07-0.79) [23]. In the present study, the incidence of bradycardia was slightly higher than in the study by George et al. [13] (12.7 versus 11%), who used a dose of 50 mg/min^-1^ phenylephrine. Ngan Kee et al. [24], using a dose of 100 mg/min^-1 ^phenylephrine, reported an incidence of bradycardia 3.3 times higher [42% (137/329)] than in our study. The results of our study suggest that the dose of phenylephrine at a rate of 50 mg/min^-1^ is more suitable for preventing maternal hypotension with a lower incidence of bradycardia.

When we stratified pregnant women according to BMI (<30 or ≥30 kg/m^-2^), we found that the incidence of bradycardia in pregnant women with a BMI < 30 kg/m^-2^ was higher than in those with a BMI ≥30 kg/m^-2^ (24.4 versus 7.2%, p=0.004). This result may be explained by the proportionally higher dose of phenylephrine that patients with a BMI <30 kg/m^-2^ received in comparison to those with a BMI ≥30 kg/m^-2^, and it suggests that we should start with an infusion of doses lower than 50 mg/min^-1 ^but equal to or greater than 25 mg/min^-1^ of phenylephrine in patients with BMI <30. This is in accordance with the recommendations of the international consensus [7]. 

The main causes of intraoperative nausea and vomiting (IONV) are maternal hypotension, pain, intravenous opioid supplementation, uterotonics, and exteriorization of the uterus by raffia (vagal reflex activation). Among these causes, spinal anesthesia-induced hypotension stands out. Hypotension may result in brain stem ischemia and consequent activation of the vomiting center. In addition, emetogenic substances such as serotonin are released from the gastrointestinal tract [25]. Studies have shown that better blood pressure control with a prophylactic infusion of phenylephrine during CS reduces the incidence of IONV [14,15,26,27]. However, in one study, despite the reduction in maternal hypotension by 50%, the incidence of IONV did not decrease [12]. In this study, 13.4% of patients had nausea, and 2.8% had intraoperative vomiting. An interesting study in which the authors evaluated IONV in groups of pregnant women who received saline, metoclopramide, or the combination of metoclopramide and ondansetron, and all patients received a 50 mg/min^-1^ infusion of phenylephrine. The incidence of hypotension was not different in the three groups (16%, 19%, and 16%, respectively) [28]. When compared to our study, they reported a higher incidence of nausea (49%, 31%, and 23%, respectively) and vomiting (15%, 10%, and 6%, respectively).

In the present study, four neonates (2.8%) had Apgar scores of <7 at the first minute, and no neonates had Apgar scores of <7 at the fifth minute. Jeon et al. [29], who included more than 3700 patients in their study, showed a significant increase in the incidence of neonates with Apgar scores <7 at the first minute in parturients who received ephedrine plus phenylephrine infusion but not with phenylephrine infusion alone. Even with the use of higher doses of phenylephrine (100 μg/min^-1^), studies did not show worsening Apgar scores [24]. 

In the present study, 6.3% of neonates had a pH <7.2. However, all neonates fully recovered without evidence of sequelae. A pH <7.2 has been used routinely to define acidaemia status. Interestingly, a recent review concludes that neither norepinephrine nor phenylephrine or ephedrine are associated with adverse neonatal outcomes or an actual increased risk of fetal acidosis using a pH <7.2 criteria [5].

The values of pH, pCO_2_, base excess, and pO_2_ of neonates' umbilical arterial blood found in our study are in accordance with other studies that used infusion of prophylactic phenylephrine at different rates [12,17,21,26]. Therefore, the use of phenylephrine infusion proved to be safe for neonates, taking into account the Apgar score and umbilical cord values. 

The prediction of risk factors could enhance clinical decision-making aiming to reduce the occurrence of hypotension during cesarean section. A prospective observational study revealed that a baseline SBP <120 mmHg was a strong predictor for the development of hypotension (relative risk ratio of 6.53) [30]. This result is in agreement with the results of our study.

To our knowledge, this is the first published prospective single-arm study in Brazil that evaluated the efficacy and safety of prophylactic infusion of phenylephrine 50 μg/min^-1^ in pregnant women undergoing elective CS under spinal anesthesia. This study is relevant because, among the anesthesiologists in Brazil, the practice of preventing maternal hypotension with phenylephrine is still little used. In addition, this study can answer that the phenylephrine dose used was adequate for obese and non-obese patients, despite the higher incidence of bradycardia in the latter.

There are some limitations in our study. First, the most important limitation of this study is that it was not designed for comparison with a control group, which weakens the power of evidence of results. Second, we included patients with chronic hypertension, which could be a potential bias. However, these patients had well-controlled preoperative blood pressure. Third, we did not use noninvasive cardiac output measurements based on impedance cardiography or transthoracic echocardiogram to assess the hemodynamic impact of the phenylephrine infusion on the patient. Lastly, the study was a single-center investigation.

## Conclusions

In conclusion, the prophylactic infusion of phenylephrine 50 μg/min^-1^ is safe and demonstrates efficacy in reducing maternal hypotension (both the incidence of hypotension and the number of hypotensive episodes), providing adequate maternal hemodynamic stability during CS under spinal anesthesia. Patients with baseline SBP <120 mmHg had a threefold increased risk of hypotension. Reactive hypertension, bradycardia, nausea, and vomiting had a low incidence. Also, the phenylephrine infusion proved to be safe for neonates, taking into account the Apgar score and umbilical cord values. The higher incidence of bradycardia in patients with a BMI <30 kg/m^-2 ^suggests the need for adjustments in phenylephrine infusion in this population. In addition, bolus administration of phenylephrine immediately prior to initiation of continuous phenylephrine infusion can further decrease the incidence of maternal hypotension. 
